# Conceptualising migraine attacks from a biopsychosocial model using qualitative and functional behavioural analysis

**DOI:** 10.1080/02813432.2023.2231034

**Published:** 2023-07-06

**Authors:** Marie Persson, Gun Rembeck, Sandra Weineland

**Affiliations:** a Primary Health Care, School of Public Health and Community Medicine, Institute of Medicine, Sahlgrenska Academy, University of Gothenburg, Gothenburg, Sweden; b Research, Education, Development & Innovation, Primary Care, Research, Region Västra Götaland, Sweden; c Närhälsan, Region Västra Götaland, Sweden; d Regional Health, Borås Youth Guidance Centre, Region Västra Götaland, Sweden; e Department of Psychology, University of Gothenburg, Gothenburg, Sweden

**Keywords:** Migraine disorders, chronic pain, pain management, qualitative research, applied behaviour analysis, biopsychosocial model

## Abstract

**Objective:**

The aim of this study was to explore patients’ experiences and management of pain in connection with a migraine attack in episodic migraine.

**Design, setting and subjects:**

This qualitative study used a semi-structured interview format based on functional behavioural analysis as commonly used in cognitive behavioural therapy. We interviewed eight participants and analysed their responses using systematic text condensation.

**Results:**

Participants’ descriptions of their experiences and management of pain from episodic migraine were sorted into three description *First physical sensations, Automatic reactions and Acts according to the interpretation.*

**Conclusion:**

From a biopsychosocial perspective, a migraine attack is much more complex than just an experience of pain. The purely biological pain prompts a number of automatic reactions leading to strategies for pain management.

## Key points

Functional behavioural analysis can increase our understanding of experiences during a migraine attack from a biopsychosocial pain perspective.

Several pain mechanisms appear to be relevant during the experience of a migraine attack than are described in the diagnostic criteria for migraine.

Pain management consists of a chain of behaviours, approaches to the migraine attack and medication and the consequences of pain management.

Knowledge and understanding of patients’ experiences of pain and pain management during a migraine attack is an important tool in the biopsychosocial model.

## Introduction

Migraine is a very widespread disease that affects people all over the world. The estimated global prevalence of migraine is 14% [1]. Migraine is a neurological disorder and episodic migraine involves migraine attacks of up to 15 days in a month [2]. Recurring pain over a three-month period is considered chronic pain, which is classified as a disease in itself [3,4]. People with three or more migraine days a month may develop chronic pain, which is seen as a consequence of migraine [5–7]. Best practice suggested for the treatment of chronic pain is diagnosis and treatment according to the biopsychosocial model [8].

Today the general practitioner (GP) plays a key role in migraine management [9]. Patients should be managed in primary care firstly, since the disease is common in the population and can be managed in primary care [10]. Guidelines emphasise that a disease such as migraine must also be managed on parallel tracks, with standard of care at the GP and more complex forms with innovative therapies in the specialist centres in the hospitals. The complex workday of a GP contains multiple clinical examinations and patients’ histories of a large variety of somatic conditions. The access to immediate imagining diagnostics is however limited. In a recent published study, it was shown that innovative machine learning (ML) as classifier for primary headache diagnoses is accurate and reliable [11].

In primary care, patients seeking for migraines have primarily been offered medical treatment. About 98% of patients with migraines take emergency medicine while only 12% use preventive medicine [12]. If patients need to take more than eight doses of emergency medicine per month, preventive drug treatment is recommended [10]. In difficult-to-treat chronic migraine, medical combination treatments of preventive measures are used [13].

The psychosocial impariment of migraine need to be adressed in treatment to promote a more integrated model of care. Migraine is associated with interictal burden among two thirds of patients. Restriction of activitys due to fear of migraine attacs, disturbed sleep and worrie of future attacs are also associated with depression among patients with migraine [14]. People with episodic migraine report higher pain catastrophising tendendcy, more feelings of hopelessness and substantial ruminative thinking than people with headaches [15]. Treatment for migraine is recommended to include preventive self-care strategies combined with both acute and long-term medical interventions [16]. Self-care strategies include exercise [17], stress-management [18], health behaviours [19] and trigger avoidance [20]. Cognitive behaviour therapy (CBT) for migraine involves different techniques, such as stress management, relaxation and cognitive strategies [12,21–23]. Finally, the role of cognitive-behavioural therapy integrated with pharmacological should be further stressed [24]. Participants who underwent CBT for migraine experienced that a non-pharmacological treatment option was positive and desirable [25]. A recent systematic review showed that the level of evidence for education and cognitive behavioural therapy for migraine remains yet low [10,12]. Educational and behavioural approaches can positively influence headache frequency and improved quality of life however, the content, duration and frequency and education formats varied widely [24]. Identifying behavioural and psychological factors associated with migraine in individuals without psychiatric comorbidities is important to improve the management of migraine through non-pharmacological strategies [15]. Holroyd et al. showed in a study that participants who were given β blocker and CBT in combination improved more regarding migraine days per month, than participants who had either only medication or only CBT [26]. This may be an indication that treating patients with migraine may benefit from a biopsychosocial perspective. The biopsychosocial model is a framework in which all factors interact and need to be considered in parallel in the treatment of migraine [27]. The long-term goal of migraine-treatment is to have fewer and milder migraine attacks, but there is different assumptions about how to accomplish this, especially when it comes to pain management.

The definition of chronic pain explains that pain is always an individual experience as in varying degree is influenced by biological, psychological and social factors [28]. Treatment for chronic pain should therefore be based on a biopsychosocial perspective [29]; however, a biopsychosocial model for migraine attacks has not yet been described. Studies have explored what it means to live with migraine [30–32], but no published qualitative studies have analysed how people with episodic migraine experience and manage pain during an attack.

To build effective treatment models focused on the core problems of living with migraine (interpretation of symptoms, perceptions of pain and pain management), qualitaitve studies are neeeded to better understand how people experience and handle pain during an attack. The current study aim to explore and describe in detail the experience and respons to a migraine attack. Historically, functional analysis has been used to explore, understand and describe problem behaviours using behavioural principles [33,34]. Functional analysis seeks to understand a specific behaviour and identify the surrounding circumstances and reinforcers that trigger and maintain the behaviour [35,36]. The current study is focused on analysing the psychological part of the biopsychosocial model and the care of people with frequent migraines in addition to the biomedical part on which the treatment has so far only been based.

### Objective

The aim of the present study was to investigate the experience and management of pain in individuals diagnosed with episodic migraine in order to produce the knowledge necessary to design effective treatment interventions for patients with migraine.

### Design

A qualitative interview study was used to explore patients’ experience of pain and pain management in patients having episodic migraine.

## Material and methods

### Context, sample and interviews

Participants were recruited via advertisement in the local newspaper and the Swedish Migraine Association. Eligible candidates were invited to participate in a qualitative interview study about their experiences of pain and pain management during episodic migraines. Twenty candidates showed interest in participating. To obtain a strategic selection with a range of variation with the participants, the following criteria were considered: rural/small town versus urban resident; single/live-in partner/married; children at home or not; college/university educated or not; employed/job seeker/on sick leave/not on sick leave and age. Inclusion criteria were patients between 18–65 years of age with a diagnosed episodic migraine in primary care. Candidates with ongoing malignant disease, for example cancer, severe mental illness, drug abuse and personality disorders, or inability to speak or understand the Swedish language were excluded. Eligible candidates were asked to answer questions about the study’s inclusion criteria in an email. Seventeen participants answered the initial questions, and three were excluded due to incorrect diagnosis. From these, the interviewer strategically chose eight participants for further interview.

Qualitative data were collected in semi-structured interviews based on functional behavioural analysis. According to DeJonckheere and Vaughn, semi-structured interviews are the most favourable method to access participants’ personal experiences, attitudes, perceptions and beliefs about a specific phenomenon [37]. Functional behavioural analysis follows an open interview guide that is at the same time flexible and leaves room to ask follow-up questions and deepen the understanding of the participants’ experiences, behaviours and their short- and long-term consequences [35,36]. The interviews were conducted in Swedish.

After initial presentation, the interview typically opened with a question such as ‘Can you tell me about a typical situation when you had a migraine attack?’ and encouraged participants to talk freely using prompts such as ‘Can you tell me more?’ and ‘How did it feel?’ The interviewer continued with the following questions when necessary: ‘What were the circumstances?’ ‘What were the first signs of a migraine attack?’ ‘What happened to you next (thoughts, emotions and physiological reactions)?’ ‘How did you manage the pain?’ and ‘What were the short- and long-term consequences of this?’. Internal validity was ensured by the interviewer asking participants repeatedly if they understood and occasionally summing up participants’ descriptions during and towards the end of the interview. The first author (MP), who had no relationship with the participants, conducted the interviews. Four interviews took place in a room at the R&D centre, two in the participant’s home and two at the workplace or university. The interviews were conducted individually, lasted up to 60 min and were recorded and transcribed by the interviewer. Travel allowance was offered to the participants who went to the R&D centre.

### Analysis

The interviews were analysed using systematic text condensation according to Malterud [38]. The second author, G.R, has expertise in qualitative analysis and was involved in planning the study. The data analysis was performed in NVivo computer software. The interviewer’s, M.Ps, preconceptions were those of a clinical psychologist and psychotherapist working with a cognitive and behavioural approach. The first author M.P, led the analytic process. To support the analytic process a team was formed with the two of the authors, M.P and S.W a clinical psychologist and associate professor. We entered the analytic process with the pre-understanding and the experience of CBT for patients with somatic conditions within the field of behavioural medicine. The analysis was conducted in four phases. Phase 1 was to read the texts to gain an overall impression related to the purpose of the study. Phase 2 involved identifying meaningful units of text and encoding them. In Phase 3, the content of each code group was sorted into sub-codes, and in Phase 4, content descriptions were created for each code group [38]. In an iterative process, main categories and subgroups were discussed, refined and defined in collaboration until consensus was reached within the analysis team. The author G.R read the results and gave her expertise comments regarding qualitative analysis.

### Research ethics

Benefits include generating new and needed knowledge, while ethical risks included the risk of a participant experiencing discomfort during the interview as they were asked to remember a typical migraine attack. The study was approved by the Gothenburg Regional Ethical Review Board (Dnr 598-15). Participants were all informed of the research in writing and orally and were to ask questions before signing consent forms. Data security was upheld.

## Results

Six women and two men aged 18 to 65 and diagnosed in primary care with episodic migraine participated in the study. Two participants used prescribed emergency medication during the typical migraine attack, three participants medicated step-wise, often starting with a non-prescription drug before proceeding to any form of triptan; two participants medicated with a non-prescription drug during the attack, and one participant did not use emergency medicine. Six participants had some form of preventive medicinal treatment. The participants had an average of two to four migraine attacks per month (Table 1).

**Table 1.** Demographic description of informants (*n* = 8).

Age, median (min–max)	52 year (36–59)
Gender	
Women (6 pt)	51 years (36–59)
Men (2 pt)	55 years (52–58)
Diagnosis	
Migraine with aura	4 pt
Migraine without aura	4 pt
Use of emergency medicine	
Prescription emergency medicine	2 pt
Prescription-free	2 pt
Both (staircase model^a^)	3 pt
No emergency medicine	1 pt
Number of migraine attacks per month, median (min–max)	2–4 (1–12)
Marital status	7 pt
In a relationship	1 pt
Married	0 pt
Live-in partner	0 pt
Divorced	
Single	
Children at home	5 pt
No children at home	3 pt
Highest education	
Upper-secondary	5 pt
College/University	3 pt
Employment	
Employed	6 pt
Unemployed	0 pt
On sick-leave, pensioner	2 pt
Occupation	
Students	1 pt
Professional within the health/social sector	4 pt
Administrative personal	1 pt
Blue collar worker	2 pt
Living urban	6 pt
Living rural	2 pt

^a^Staircase model; informants medicated step-wise, often starting with a non-prescription drug before going on to some form of Triptan if the first measure was insufficient.

### Main categories

The eight participants’ experiences and management of pain from episodic migraine are described under three descriptions, *First physical sensations, Automatic reactions* and *Acts according to the interpretation*, each composed of smaller, but related, subgroup (Table 2).

**Table 2.** Overview of the systematic text condensation according to Malterud.

Meaning units	Code group	Subgroup	Description
Pure biological pain	Centrally located pain and complex pain pattern during the migraine attack	Localisation Character Pain level	First physical sensations
Automatic reactions	Thought content	1. Loss of control experienced with aura A. Restriction (cannot drive) B. Fear of severe migraine attacks, Fear of damage to vision C. Aura was associated with unpleasant symptoms such as nausea and loss of sensation. 2. Interpretations 3. Thoughts on cause 4. Fear that something is wrong 5. Responsibility and guilt 6. Fear of what others will think	Automatic reactions
Pain management	Pain management – a chain of sequences	Behaviours Approach to the migraine attack and medication Consequences of pain management	Acts according to the interpretation

### First physical sensations

#### Localisation

Most participants reported experiencing pain beginning as pressure at the height of the forehead, but further inside the head: ‘The pain is central to the head’ (Participant [P] 2) ‘… in the middle of the head [it] is too large, it pushes’ (P 7). All felt that the pain wandered during the attack and became worse when it approached the forehead and the eye.

#### Character

The pain was characterised by most participants as pulsating, throbbing, stinging, stabbing, burning, cutting and oppressive. The pain could start as throbbing and then become cutting. Pulsating was associated with worse episodes: ‘… it can pulsate then, of course, when I have those horrible attacks, too’ (P 1), and ‘It changes character from that grinding and then it becomes more oppressive and then it becomes more burning’ (P 6).

#### Pain level

The participants described the pain as mild to severe. Those who experienced ‘aura’ described their pain growing during or after the aura, but also as being manageable. As one participant said, ‘You can go […] a whole day and work and be running and driving and be involved, but it’s this flicker that makes me unable to get home as well’ (P 5). Others described the pain as severe and intolerable pain (P 4).

### Automatic reactions

#### Aura and loss of control

Half of the participants reported visual auras as the first symptom of a migraine attack. Auras were described as flickers of light, little moons, or lightning that grew bigger and moved further away into the periphery or like a shaky black and white film whose strobe effect creates an uncomfortable feeling of stress. The aura could change shape and meaning on different occasions. One participant described a triple aura that signalled the return of both aura and pain on three consecutive days. Participants who experienced aura also felt a loss of control and some felt the aura was more unpleasant than the pain.

Aura was associated with loss of control in three areas: insecurity and unpleasant symptoms; loss of sensation and nausea, fear of severe migraine attacks and permanent damage to the eyes and the inability to drive home or do anything other than go to bed: ‘it is as if you get a small damage to the retina every time’ (P 5), ‘I get so limited, it gets so definite, I cannot do anything but […] go to bed’ (P 5).

#### Interpretations

Most participants experienced the first physical symptoms early in the morning and some during the day. Those who did not experience aura interpreted centrally located pain, a taste in their mouth, a creeping sensation on their skull and body and increasingly intense pain and nausea as their first physical experiences of migraine:

When I start to feel that pressure then I take three naproxen… and it has actually happened sometimes that it has not wandered on then and become [a migraine], and sometimes it has done so anyway, and then I take [zolmitriptan]. When I have come to the saw blade position… then I give up. (P 7)

Some felt it was easy to discriminate between ordinary headaches and migraines by their location and pattern, with ordinary headaches thought to be located more superficially:

The tension headache is more on the outside, around the whole head, so to speak, and this one, which I think basically always turns into a migraine, does not sit as pressure from the outside, but it is as if someone has a fist in the middle of my brain and like, *shhh*, presses somehow. (P 6)

Participants described other headaches as different to those that usually develop into migraines: ‘Then it sits more in the back of the head, like a hat, as well as over the entire head, more grinding’ (P 3).

#### Thoughts on causes

When participants experienced symptoms of migraine, they sought internal and external causes and came to different interpretations of what might have provoked the attack. They considered factors such as hormonal cycles, what they’d eaten or drunk, feelings that needed to be processed or expressed, fatigue, and exhaustion: ‘It starts when I’m sleeping, usually. And that’s because I’m tired’ (P 1): ‘So there’s something that wants out. Some feeling I need to feel. And then it’s sometimes so hard, it’s so painful, that it kind of kicks off a migraine attack along the way. So I’m pretty much thinking’ (P 7).

#### Fear that something is wrong

The participants’ experiences of pain made them worry that something was breaking in their head, that the pain was something more than migraine, and could be something worse such as a brain haemorrhage: ‘When the medicine doesn’t take, what if it’s something else?’ (P 2) ‘[A] sharp pain will just slam into my head and then I think I no longer exist’ (P 5).

#### Responsibility and guilt

Participants described having self-critical thoughts during the attack about how they could have exposed themselves to the migraine, what they could have done differently, and whether they had done something wrong.

There were thoughts of being more attentive to the pre-symptoms and menstrual cycle, hoping to be even faster to prevent the attack, make any action not get migraines: ‘I think I can learn to find even earlier warning signs and take some action, then maybe I would not get migraine attacks’ (P 3). One participant said: ‘that it is not because I behave wrong, it is not self-chosen. I’m trying to manage my life’ (P 6). While others blamed themselves: ‘I often blame myself because I know it’s when I’ve put too much pressure on myself’ (P 8), and ‘I took that glass of wine or I ate that chocolate and so on… ’ (P 2).

#### Fear of what others will think

Participants described memories of disabling interactions with significant others, during the attack and or when they were migraine-free. Participants had been told that people with migraines need control, are perfectionists or are depressed, and they often internalised these assessments that accused them of their own suffering: One felt that if they ‘were just different in some way, I would not have this’ (P 6), and another thought that others judged her, and asked ‘Is she sick again?!’ (P3).

### Acts according to the interpretation

#### Behaviours

Common behaviours in participants were to take it easy, take medication, eat something, find a distraction, wait out the pain and try to asleep:

Then I took everything. Nasal spray and this new medicine. I didn’t have a syringe, but I took the tablets, imigran and citodon. I took off everything I had on [… and] spread out during the day. I’m kind of trying to find something [medicine] that breaks the cycle of the attack. (P 2)

The participants with the least pain during the attack experienced aura, made a quick interpretation and responded immediately to the first physical sensations. A participant did not take any medicine with the aim of curing the attack:

Now the body has to heal. I don't give a damn about taking medication, just take care of myself, and then I know from experience that I will, when I get well, be really good. When I take medicine, I become functional but not restored. (P 6)

#### Approach to the migraine attack and medication

When participants experienced the first physical sensations of migraine, they hoped they misunderstood the symptoms and that they would disappear on their own:

I start to see that I’m having a hard time seeing the letters… and my first, my immediate, absolutely first reaction is, ‘No, it’s not a migraine!’ And then I keep writing. But then, then I realise, no, I don’t really see anything… and then I will be, that’s very strongly associated with anxiety for me… it’s about minutes, maybe one or two [from the start of physical sensations to taking emergency medication. (P 3)

Some experienced fear of taking too much medicine during the attack. When the migraine attack came early in the morning, participants quickly took emergency medicine to be able to go to work: ‘So I have broken through many attacks by taking medicine at four or five o’clock in the morning, and then waking up at half past six able to get up and go to work’ (P 2).

#### Consequences of pain management

The attacks lasted for one to five days. The emergency medicine did not seem to change the pain at the moment, but in the slightly longer term it decreased and returned: ‘Then [in severe pain], I usually think, honestly, if there was the option to cut off my head but still live a decent life, then I’d do it’ (P 3). The long-term consequences of the migraine attack were lost sharpness (mental sharpness) and feelings of helplessness and despair: ‘I think I have lost a little sharpness, I think I’ve got a little affected or tough in some way…. The pace in the body slows down, you become reduced, you feel sick… ’ (P2): ‘I think it’s more a resignation… a lack of power…. Now it’s coming again. You hope every time it’s over that it’ll never happen again. But it’s like… you’re trapped in this migraine body in some way… powerlessness and helplessness’ (P 7).

## Discussion

This study showed, that a migraine attack is much more complex than just an experience of pain. The biological pain initiates an number of automatic reactions and strategies for pain management. In the diagnostic criteria for episodic migraine, only pulsating pain and semi-lateral location is mentioned [2]. Research studies describing the character and localisation of pain during a migraine attack show a more varied picture [39–41]. In this study, most participants described the pain as a centrally located pressure in the head. The various reported pain characteristics are consistent with the operationalisation of migraine as a mixed pain [5], with several current pain mechanisms, such as the fact that many of the migraine attacks started spontaneously at rest early in the morning [5,42]. Participants with episodic migraine described their experiences of the intensity, duration and course of the pain in line with the results from previous research on the natural course of the migraine attack [43].

The body experiences and interprets pain as a threat [42]; we then must choose between different actions to protect ourselves. Automatic reactions include interpretations of symptoms, consideration of the best course of action and possible consequences of the situation. Aura has previously been described in research [44], but few studies describe its meaning to the individual sufferer of a migraine attack. The participants associated aura with loss of control in three different areas and interpreted it as a signal that a migraine attack was imminent.

Several hypotheses posit various causes of migraines [45–47]. An automatic reaction common to our participants during a migraine attack was to look for its cause. Identifying the cause of a negative event is likely to have a survival function for the individual [48]. The participants’ experience of discomfort and anxiety during the attack and their fear that something was wrong is consistent with the function of acute pain, which is to warn of tissue damage and prompt protective action [42].

Participants in the current study described fear of what others will think. According to the biopsychosocial model migraine attacks occur in a social context. How one is perceived in the group has an impact on an individual’s view of themself. Shame and self-criticism were prominent automatic reactions during the migraine attack. Research shows that shame and guilt is common in people who feel they cannot control their pain [49]. Fear of what others will think is also in line with previous studies that have shown that patients with migraine are worried about being misunderstood [31]. Patients with migraines perceive that society generally tends to dismiss migraine as a ‘common headache’ and to not understand the real impairment caused by migraines [31]. More studies with a qualitative design, could shed further light into the social effects of suffering from frequent migraine.

**Figure 1.**
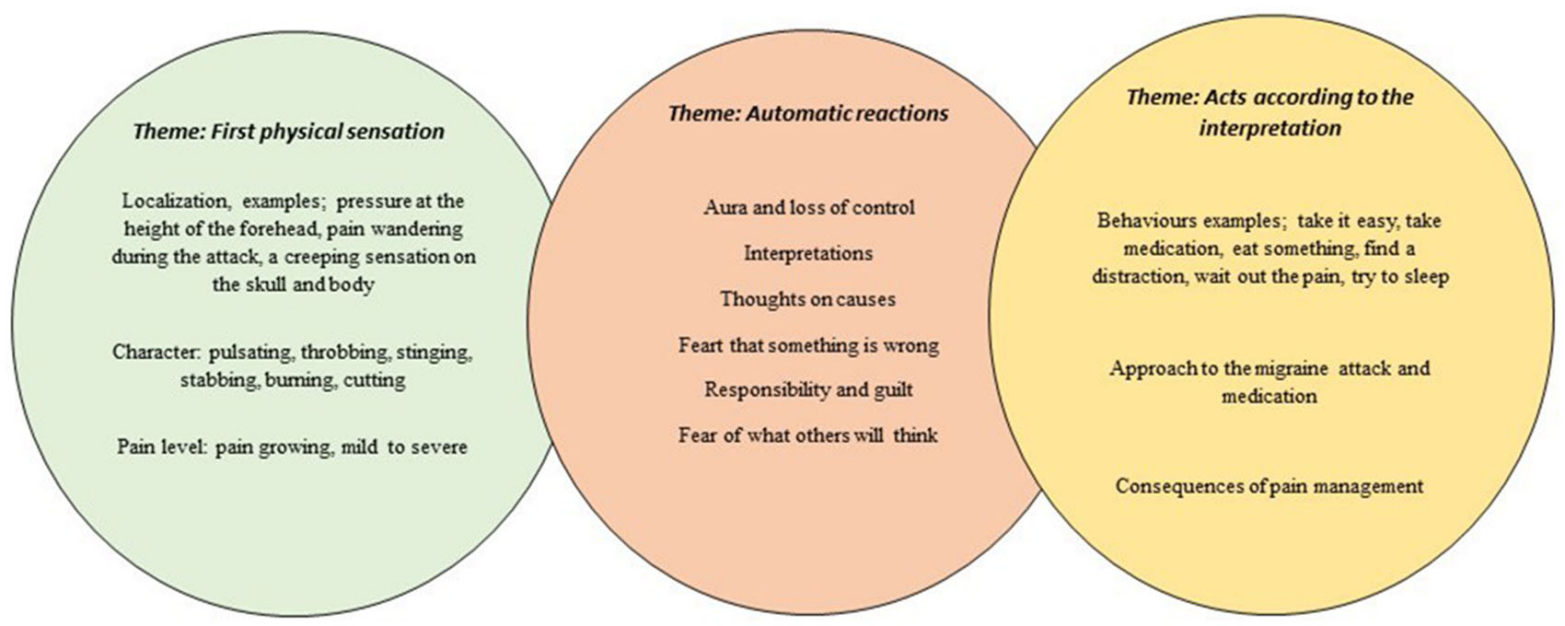
Conceptualisation of the experience of pain and management of a migraine attack.

Pain management consists of a chain of sequences such as physical sensations, automatic reactions and acting according to the interpretation (see Figure 1).

Participants with aura generally recognised migraine easily and interpreted the aura as the first sensation of an imminent migraine attack. Thoughts that guided most of the participants in pain management seems to be to take emergency medication at an early stage of the migraine attack. A couple of participants waited up to a few hours before taking emergency medicine. If the first symptoms came early in the morning, they took emergency medication to be able to go to work and if the first symptoms came during the day, several considerations were made [50]. The participants were medicated with various emergency medications, received some relief during the attack but were never completely pain-free. Although there are medical guidelines for migraines, they are interpreted differently by patients and caregivers, the implementation and reception of guidelines is complex, as partly revealed in this study focusing on pain management in migraine attacks.

To date, few theoretical models of migraine management explore the functions of behaviours. The term ‘function’ describes the role, purpose and expected outcome of a behaviour. Previous studies showing that patients are dissatisfied with their emergency medication [51] and that adherence to preventive medication is low and difficult to predict [52,53]. Insufficient treatment of that underlying disease will lead to the secondary development of chronic pain [54]. As the current study mapped, there are possible reinforcing factors in the form of automatic reactions and choice of pain management strategies that can be important to recognise and understand when dealing with and treating migraines. The new knowledge that the current study has provided, points out how we can develop and further build a treatment strategy of frequent migraine, for example handling the feelings of despair and helplessness, loss of control and thus reduce the discomfort and anxiety experienced by people suffering from migraine.

In conclusion, the experience of pain and pain management in episodic migraine is complex and individual. The functional analysis described some common patterns in the form of first physical sensations, automatic reaction and action on interpretations that can to varying degrees influence the experience of pain and pain management in migraines. The influence of psychological factors on pain and pain management during a migraine attack with an underlying neurological disease need to be further explored.

## Limitations

Limitations of the current study include the small sample, however generalisability is not the aim of qualitative research. The more usable data that are collected from each person, the fewer participants are needed in the qualitative analysis. We consider the material to be richly textured and that a degree of saturation was achieved in the interviews. It is however important to notice that each person’s pain management can look different over the life cycle, and the medical and societal norms of pain management can also change over time.

In further studies, the influence of social and psychological factors on pain and pain management during a migraine attack needs to be investigated. It would also be good to test whether a psychological treatment program containing interventions from a pain perspective as well as improving aspects of living with migraine can prevent and reduce the progression to frequent and chronic migraine.

## Contribution to policy and practice

Functional behavioural analysis is a helpful tool to increase understanding of migraine experiences from a biopsychosocial pain perspective. It can be used to clarify and increase the knowledge of what happens to a patient during a migraine attack, that person’s approach to pain management and the short- and long-term consequences of those experiences and reactions. We hope that patients will feel safer and less alone in their experience if it can be communicated to other important people. This increased knowledge should also allow professionals at the clinic to form hypotheses and develop tools to facilitate functional pain management.
